# Cochlear and Bone Conduction Implants in Asymmetric Hearing Loss and Single-Sided Deafness: Effects on Localization, Speech in Noise, and Quality of Life

**DOI:** 10.3390/audiolres15030049

**Published:** 2025-04-27

**Authors:** Oana Astefanei, Cristian Martu, Sebastian Cozma, Luminita Radulescu

**Affiliations:** 1 Doctoral School, Grigore T. Popa University of Medicine and Pharmacy, 700115 Iasi, Romania; oana.botezatu-astefanei@d.umfiasi.ro; 2 ENT Clinic Department, Clinical Rehabilitation Hospital, 700661 Iasi, Romania; martu.cristian@umfiasi.ro (C.M.); sebastian.cozma@umfiasi.ro (S.C.); 3 Department of Otorhinolaryngology, Faculty of Medicine, Grigore T. Popa University of Medicine and Pharmacy, 700115 Iasi, Romania

**Keywords:** single-sided deafness (SSD), asymmetric hearing loss (AHL), cochlear implant (CI), bone conduction implant (BCI), binaural hearing, localization, tinnitus suppression, self-reported outcomes

## Abstract

**Background**: Single-sided deafness (SSD) and asymmetric hearing loss (AHL) impair spatial hearing and speech perception, often reducing quality of life. Cochlear implants (CIs) and bone conduction implants (BCIs) are rehabilitation options used in SSD and AHL to improve auditory perception and support functional integration in daily life. **Objective**: We aimed to evaluate hearing outcomes after auditory implantation in SSD and AHL patients, focusing on localization accuracy, speech-in-noise understanding, tinnitus relief, and perceived benefit. **Methods**: In this longitudinal observational study, 37 patients (adults and children) received a CI or a BCI according to clinical indications. Outcomes included localization and spatial speech-in-noise assessment, tinnitus ratings, and SSQ12 scores. Statistical analyses used parametric and non-parametric tests (*p* < 0.05). **Results**: In adult CI users, localization error significantly decreased from 81.9° ± 15.8° to 43.7° ± 13.5° (*p* < 0.001). In children, regardless of the implant type (CI or BCI), localization error improved from 74.3° to 44.8°, indicating a consistent spatial benefit. In adult BCI users, localization error decreased from 74.6° to 69.2°, but the improvement did not reach statistical significance. Tinnitus severity, measured on a 10-point VAS scale, decreased significantly in CI users (mean reduction: 2.8 ± 2.0, *p* < 0.001), while changes in BCI users were small and of limited clinical relevance. SSQ12B/C scores improved in all adult groups, with the largest gains observed in spatial hearing for CI users (2.1 ± 1.2) and in speech understanding for BCI users (1.6 ± 0.9); children reported high benefits across all domains. Head shadow yielded the most consistent benefit across all groups (up to 4.9 dB in adult CI users, 3.8 dB in adult BCI users, and 4.6 dB in children). Although binaural effects were smaller in BCI users, positive gains were observed, especially in pediatric cases. Correlation analysis showed that daily device use positively predicted SSQ12 improvement (r = 0.57) and tinnitus relief (r = 0.42), while longer deafness duration was associated with poorer localization outcomes (r = –0.48). **Conclusions**: CIs and BCIs provide measurable benefits in SSD and AHL rehabilitation. Outcomes vary with age, device, and deafness duration, underscoring the need for early intervention and consistent auditory input.

## 1. Introduction

Single-sided deafness (SSD) is defined as severe-to-profound sensorineural hearing loss (SNHL) in one ear while normal hearing is preserved in the contralateral ear. Asymmetric hearing loss (AHL) is characterized by an interaural threshold difference of ≥15 dB hearing loss (HL) between the ears, with the better ear threshold ranging from 30 to 55 dB, as determined by the pure-tone average of four frequencies (PTA4), and one ear presenting SNHL [1,2,3].

The auditory cortex integrates input from both cochlear nerves, processing sound intensity, duration, and frequency differences, which are essential for binaural hearing [4,5,6]. This central processing creates a three-dimensional perception of sound, enabling spatial localization and improved speech perception in noisy environments through binaural summation and the squelch effect [4,5,6]. In normal-hearing (NH) individuals, redundant auditory input from both ears eases the suppression and summation of signals, providing an advantage in speech processing in complex environments [4,5,6]. Sound localization relies on interaural time differences (ITDs) for low frequencies (<1500 Hz) and interaural level differences (ILDs) for high frequencies (>1500 Hz) [5]. In patients with SSD and AHL, localization is significantly impaired due to the absence of binaural cues, leading to increased azimuth estimation errors [1,2,4,5,6]. These individuals are unable to separate sound from noise when spatially distinct sources are present (the squelch effect) and lack binaural summation due to unilateral input loss. The head shadow effect further obstructs sound transmission to the hearing ear, compromising auditory perception [4,5,6]. Consequently, monaural hearing induces cortical auditory modifications and adaptive changes in signal processing, as binaural mechanisms are disrupted [4,6].

The ability to perceive and understand speech in complex environments, localize sound sources, and filter background noise is crucial for daily functioning, particularly in social interactions and communication. When auditory input from one ear is compromised, these processes become significantly impaired, leading to increased listening effort, auditory fatigue, and difficulties in communication, ultimately affecting social engagement and professional performance [7].

Until the early 2000s, normal or near-normal hearing in one ear was considered sufficient for effective communication. However, research has since proven the significant and often-underestimated impact of asymmetric hearing loss. Unlike other sensory impairments, the effects of asymmetric hearing loss are less noticeable, which leads to the under-recognition of individuals’ needs.

To address these challenges, various auditory technologies have been developed, including implantable hearing devices such as cochlear implants (CIs) and bone conduction implants (BCIs) [2,8,9,10,11,12,13,14]. Cochlear implantation restores auditory perception in individuals with SSD and AHL by directly stimulating the cochlear nerve, bypassing damaged hair cells, and re-establishing neural input to the auditory cortex [4]. This intervention enhances binaural cues, mitigates the head shadow effect, improves spatial hearing, and helps speech perception in noise, provided the auditory nerve remains functional. Although CI users generally exhibit reduced localization ability compared to normal-hearing individuals, adaptation occurs over time [15,16,17]. These findings suggest that true binaural hearing is achievable only with a CI, despite its first indication being for intractable tinnitus in the affected ear. Severe tinnitus (rated 6–7 out of 10) was a primary factor in implant choice, as CIs are believed to not only restore binaural hearing, but also alleviate tinnitus perception [18,19]. Cochlear implantation in adults with SSD or AHL significantly improves speech perception, tinnitus control, sound localization, and quality of life (QOL) [20,21,22,23,24,25,26].

Bone conduction implants serve as an alternative treatment, particularly for SSD patients who are not CI candidates [11,12,13,14]. These devices reroute auditory signals through bone conduction, improving sound awareness and reducing the head shadow effect. While BCIs may enhance speech recognition in noise, they do not restore binaural hearing, and their impact on sound localization is limited. In patients with SSD, a BCI is used in a CROS configuration (BCI-CROS), where sounds from the deaf side are transmitted to the normal-hearing cochlea. Although this input reaches the same auditory pathway with a slight delay, it enhances access to environmental sounds without reactivating the impaired side, making it a suitable option when cochlear implantation is contraindicated. However, BCIs are recommended for a subset of patients, particularly those prioritizing speech perception in noise, but who are ineligible for a CI due to hearing loss characteristics (e.g., onset, duration) or personal preference [27,28,29].

An essential aspect of SSD and AHL management is finding the etiology of hearing loss. Various causes include inner ear anomalies, head trauma, Ménière’s disease, vestibular schwannoma, ischemia, autoimmune disorders, and infections; however, the etiology of hearing loss often remains unknown [30]. Management approaches differ significantly between congenital or early-onset cases and those who acquire hearing loss after language development—whether in children or adult—highlighting the importance of accurately determining the onset to guide appropriate intervention strategies [21]. Idiopathic post-lingual sudden SNHL is one of the conditions that benefits from CIs. In congenital/early-onset SSD, caution is necessary when selecting rehabilitation solutions (CI, BCI, CROS), as cochlear nerve deficiency (CND) or cochlear nerve hypoplasia appears to be the most prevalent cause [31]. Since cochlear nerve function is a key prognostic factor for CI success, proper candidate choice is imperative [2].

The most common etiology of pediatric SSD is cochlear nerve deficiency, although the condition itself remains relatively rare. High-resolution temporal bone imaging is essential for assessing cochlear implantation candidacy [32,33]. When cochlear implantation is contraindicated due to cochlear nerve deficiency or severe inner ear malformations, bone conduction implants (BCIs) may remain anatomically viable, providing an alternative rehabilitative strategy in selected cases. A systematic review and meta-analysis found that cochlear implantation in children with SSD was associated with clinically meaningful improvements in audiological and patient-reported outcomes, with shorter durations of deafness linked to better outcomes [34].

The eligibility criteria for cochlear implantation in adult-onset SSD populations continue to evolve, with several previous contraindications now being reconsidered [2,35,36]. The duration of auditory deprivation remains a major point of discussion. The relationship between the duration of auditory deprivation and implant outcomes has been extensively documented. For instance, a study from 2013 demonstrated a significant inverse correlation between the duration of deafness and speech recognition performance in a large cohort of adult CI users, with the most pronounced declines observed after 10 years of deprivation [37]. The inherent variability among patients needs a nuanced approach, recognizing individual ability to tolerate and integrate CI technology. This underscores the importance of comprehensive evaluation in deciding CI candidacy and planning interventions [2,31,35,36,38].

When comparing CIs with BCIs, discrepancies become clearer in noisy listening environments [39,40]. A meta-analyzer had shown that CIs provide substantial advantages in sound localization, tinnitus suppression, and overall quality of life assessment, whereas BCIs perform better in speech discrimination in noisy settings and in assessing quality of life related to background noise perception [13].

Regarding MRI compatibility, cochlear implants are generally MRI-conditional up to 1.5 T or 3 T, depending on the model and safety measures. For bone conduction systems, active transcutaneous implants allow 3 T imaging without magnet removal, while percutaneous devices typically have lower compatibility and may cause imaging artifacts due to their direct skin penetration.

The implementation of auditory technologies can significantly enhance the quality of life for individuals with SSD and AHL, aiding social and professional integration [41,42,43]. Regarding the benefits of these devices, the recent consensus on SSD highlights three essential outcome domains: the impact on social situations, group conversations in noisy environments, and spatial orientation [44]. These recommended outcome domains serve as a minimum standard but do not restrict clinical studies from evaluating additional measures. Five other outcome domains—listening effort, one-on-one conversations in general noise, awareness of sound, device usage, and listening in complex situations—were also named in the final consensus session. The primary outcome domains align with the first consensus group’s recommendations [1], which served as the foundation for the present study, alongside five other secondary measures.

## 2. Materials and Methods

This prospective observational study was conducted at the ENT Clinic, within the Department of Otorhinolaryngology and the Audiology Department, with the aim of evaluating auditory rehabilitation outcomes in both adult and pediatric patients with SSD and AHL. This study included patients eligible for auditory implants, such as CIs and BCIs, based on the specific indication and contraindication of each device (patients with prolonged deafness or congenital perilingual hearing loss received BCIs). This selection strategy also reflects differing cortical implications: while a CI reactivates the previously deprived auditory pathway, a BCI (in a CROS setup) leverages the intact pathway, potentially avoiding the effects of cortical reorganization observed in long-term asymmetry.

The study method (the AHL/SSD audiological criteria and outcome measures) follows the recommendations of the unified framework for SSD/AHL studies published in 2017 by the international consensus group [1] and is aligned with the approach used in recent long-term outcome evaluations, such as the study by Távora-Vieira et al. (2019) [45].

Device selection was a collaborative process involving both physician recommendations and patient preferences, guided by national guidelines and manufacturer recommendations, to ensure the best, individualized treatment approach. Pre-procedural counseling was conducted by a multidisciplinary team to establish realistic expectations and support informed decision-making. Interventions were chosen based on clinical and audiological characteristics, with treatment decisions tailored to each patient’s audiologic diagnosis, individual pathogenesis, and the duration of hearing loss. In alignment with individualized care principles, randomization was not applied. This personalized approach not only enhanced the relevance of this study by reflecting real-world clinical practice, but also ensured ethical integrity by prioritizing patient autonomy in the decision-making process.

Patient outcomes were systematically evaluated following rehabilitation to assess the effectiveness of the selected interventions. Participants, all native Romanian speakers with speech recognition <60% at 60 dB SPL in the poorer ear, were grouped based on the following clinical and audiological criteria:

SSD: hearing loss of at least 70 dBHL in the affected ear with an audiometric threshold of ≤30 dBHL in the contralateral ear.

AHL: pure-tone average (PTA4) thresholds of 70 dB in the poorer ear and between 31 and 55 dBHL in the contralateral ear.

All patients underwent imaging evaluations using MRI or CT to rule out anatomical anomalies and confirm their eligibility for cochlear implantation or bone conduction implantation. These investigations enabled the assessment of auditory nerve presence, exclusion of auditory nerve tumors, identification of progressive lesions, and evaluation of anatomical compatibility with the selected device. MRI was primarily used in patients with sudden or progressive sensorineural hearing loss to exclude retrocochlear pathologies, such as vestibular schwannoma, and to confirm the presence of the cochlear nerve. In these cases, imaging played a crucial role in determining the indication for implantation, ensuring that the auditory structures were suitable for electrical stimulation. CT was conducted to evaluate the condition of the temporal bone and cochlear anatomy, which is necessary for assessing structural compatibility for implantation. For BCI candidates, this imaging modality allowed the evaluation of temporal bone thickness and the identification of potential anatomical variations that could influence device placement.

All implant decisions in this study were made according to the criteria set by the National Cochlear Implantation Program. In line with these guidelines, device selection was based on clinical, audiological, and imaging data, including the onset and duration of deafness, speech understanding, and anatomical findings on CT or MRI. In the SSD group, all patients met the inclusion criteria for the affected ear, while the contralateral ear provided preserved hearing and speech understanding. Imaging was systematically performed to confirm cochlear nerve presence and anatomical suitability for implantation. While electrophysiological tests, such as electrically evoked auditory brainstem responses (eABRs) and electrically evoked auditory late responses (eALRs), were not applied in this cohort, they may be valuable in future clinical protocols to support implant candidacy decisions, particularly in patients with uncertain neural integrity and functionality. As demonstrated in recent studies, eABR may also be used intraoperatively to verify auditory nerve activation and assess functional electrode placement, especially when neural response is uncertain preoperatively [46,47]. Additionally, OTOPLAN software version 3.1.0 was selectively used for individualized surgical planning in CI and BCI candidates, to secure the placement of the hearing implant in relation to sensitive anatomical landmarks and to optimize audiological outcomes [48].

Outcome Measures

Baseline (T0) Assessments:

Pure-tone audiometry (PTA4): hearing thresholds measured at 0,5, 1, 2, and 4 KHz.

Localization accuracy: sound source localization.

SSQ12A scale: speech, spatial hearing, quality of hearing (0–10 scale).

Tinnitus severity: visual analog scale (VAS, 0–10).

Follow-up (T1) assessments were conducted once for each patient, between 12 and 24 months after implant activation.

PTA4: aided for poorer ear and unaided/aided for better ear in AHL thresholds.

Speech-in-noise tests (SRT50): Assessed head shadow effect, release from masking, binaural squelch, and summation. Aided and unaided conditions.

Localization accuracy: aided and unaided conditions.

SSQ12B/C: post-intervention quality of hearing changes (−5 to +5 scale).

Tinnitus impact: aided and unaided assessment via VAS.

The estimated duration of device use was based on self-reported data from patients.

Audiological Evaluation and Hearing Thresholds

The audiological assessment for implantation candidacy included measuring pure-tone audiometry thresholds across frequencies ranging from 0.25 to 8 kHz for each ear in a standard audiometric booth. At T0, word recognition scores were taken as the percentage of correctly identified words at 60 dB SPL. Equinox 2.0 audiometer software, version 2.12.3 (Interacoustics^®^, Denmark), was used for conducting pure-tone and speech audiometry. TDH39 headphones were used for pure-tone thresholds and speech recognition tests. During follow-up appointments, PTA thresholds in the better ear were monitored to ensure compliance with SSD and AHL criteria at T1. For AHL subjects using a conventional hearing aid in the contralateral ear, assessments were conducted while aided. Audiological gain at T1 was assessed using free-field stimuli from a loudspeaker placed 1 m in front of the subject, with the better ear masked or occluded.

Speech Perception in Noise Assessment

Speech perception in noise was evaluated using a speech-in-noise test (Interacoustics^®^ A/S, Middelfart, Denmark with synthetic sentences, each containing five semantically unpredictable words. This test measured SRT50, defined as the signal-to-noise ratio (SNR), in dB at which the subject correctly recognized 50% of the presented stimuli. This study used a sentence score, with 50% of sentences correctly recognized. In training sessions, participants were presented one list in quiet conditions and another list with noise at SNR 0. The lists of 10 sentences were randomly presented and designed to have similar intelligibility. The background noise was set to 65 dB SPL, while the speech level was modified according to subject responses. The tests were performed under the following presentation configurations:

S_0_N_0_: speech and noise are presented from a single front-facing loudspeaker.

S_0_N_SSD/AHL_: speech presented from the front, noise from the deaf side.

S_SSD/AHL_N_AH_: speech presented from the SSD/AHL side, noise from the contralateral acoustic hearing (AH) side.

The sentence test was administered both with the device activated and without it at T1. Any positive difference in dB between the unaided and aided condition indicates an improvement. Improvement was calculated using the following parameters:

Head shadow effect: SRT S_SSD/AHL_N_AH_ unaided/SRT S_SSD/AHL_N_AH_ aided.

Release from masking: SRT S_0_N_0_ unaided/SRT S_0_N_SSD/AHL_ aided.

Binaural squelch: SRT S_0_N_SSD/AHL_ aided/SRT S_0_N_SSD/AHL_ aided.

Binaural summation: SRT S_0_N_0_ unaided/SRT S_0_N_0_ aided.

Sound Localization Evaluation

The accuracy of sound localization was evaluated using a setup with 7 speakers placed in a semicircle spanning 180°, with each speaker positioned 30° apart. Participants were directed to maintain head stability, and responses were assessed in terms of localization error and bias, both measured in degrees. The stimuli consisted of randomly presented white noise bursts, each with 20 ms rise and fall times. Two noise signals (unfiltered and filtered CCITT sound) were randomly presented at 65, 70, and 75 dBA for each speaker. Each stimulus was presented once, totaling 42 presentations (7 speakers × 3 levels × 2 signals). Each listening condition test lasted approximately 2–3 min. The localization measures analyzed were the root mean square (RMS) error between the indicated and actual speaker angles. An RMS error ≤15° is considered normal for hearing. A lower RMS error indicates better localization accuracy. Localization measurements took place in a semi-anechoic chamber with sound-attenuating walls and flooring for free-field testing. The average background noise level in the chamber was 41.05 dBA, as measured using a PCE-322A sound level meter (measurement range: 30 dB to 130 dB, resolution: 0.1 dB). The same chamber was used for the SRT50 evaluation.

Speech, Spatial, and Qualities of Hearing Scale (SSQ12)

The Speech, Spatial, and Qualities of Hearing Scale (SSQ12) consists of three subscales evaluating speech understanding, spatial hearing, and sound quality, with a scoring range from 0 (unable to perform the indicated action) to 10 (perfect hearing perception). Subscale scores provide insights into the specific disabilities most affected by asymmetric hearing. The overall SSQ score was determined by averaging all the responses provided. SSQ12B and SSQ12C assessed self-reported changes after rehabilitation intervention, with scoring ranging from −5 to +5, allowing the identification of improvements or declines in hearing-related quality of life. The questionnaires were filled out in paper format. Participants could request clarification from the research team. Evaluated parameters included total SSQ12A score, total SSQ12B/C score, and subscale SSQ12B/C scores (speech, spatial, qualities).

Tinnitus Assessment

Tinnitus severity was measured in both CI and BCI subjects using a visual analog scale (VAS). A score of 10 indicated maximum disability, while 0 signified no tinnitus-related handicap.

Statistical analyses were conducted in Python using descriptive and inferential methods to evaluate auditory rehabilitation outcomes. Patients were grouped by hearing loss type (SSD or AHL), and subgroup analyses were performed separately for adults and children where appropriate. Tinnitus analyses were restricted to adults.

Parametric tests (e.g., *t*-tests, ANOVA, ANCOVA) and non-parametric alternatives (e.g., Wilcoxon signed-rank test, Mann–Whitney U test) were selected based on data distribution. The significance threshold was set at *p* < 0.05. Post hoc effect sizes (Cohen’s d) and statistical power (1–β) were computed for each comparison to assess the robustness of observed effects. Bonferroni correction was applied for multiple comparisons when necessary. Estimations were performed using Stata MP 16 and Python 3.13 software packages.

## 3. Results

### 3.1. Patients

In the CI-implanted adult group (n = 18), 13 patients had SSD (mean age: 42 years), and 5 had AHL (mean age: 44.2 years, subject 14–subject 18). In the BCI-implanted group of adults, five patients had perilingual, possibly congenital, hearing loss (mean age: 32.8 years), while four had postlingual hearing loss (mean age: 50.7 years). A pediatric cohort (n = 9, age range: 9–16 years) was also included, comprising five patients who received a CI for postlingual hearing loss and four who received a BCI for perilingual hearing loss. Table 1a,b present the demographic characteristics of the patients and the underlying causes of their hearing loss. The results indicate that CI recipients had a significantly shorter duration of asymmetric hearing loss before implantation compared to BCI users. On average, CI users had lived with hearing asymmetry for 5.4 years, while BCI users had a mean duration of 19.5 years. In pediatric patients, implantation occurred significantly earlier (mean: 12.7 years).

Although not used as a formal grouping variable, clinical history showed varied pre-implant auditory stimulation. Most adult SSD patients reported sporadic and unsatisfactory hearing aid use, and none had used a CROS device. Among the BCI users with congenital or perilingual onset, only two had trialed hearing aids without sustained benefit. AHL patients demonstrated more consistent hearing aid use, especially in the better ear, with four still using one at the time of evaluation. These observations provide relevant context regarding prior auditory input.

### 3.2. OUTCOMES

#### 3.2.1. Pure-Tone Audiometry (PTA4)

**Figure 1:**
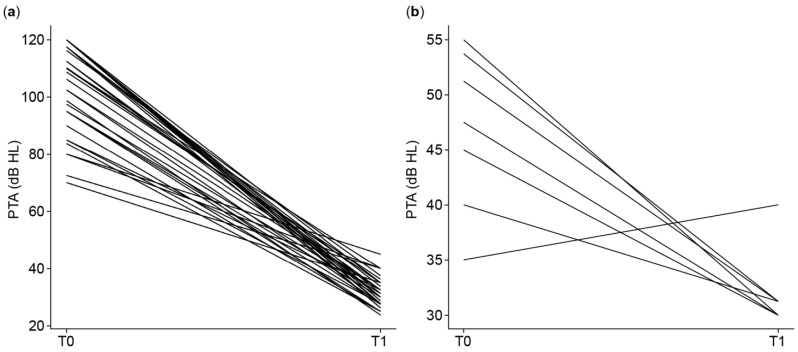
PTA in the poorer (implanted) ear at baseline and follow-up (**a**). PTA in the better ear (AHL subjects) at baseline and follow-up (**b**). (**a**) Individual PTA4 thresholds (dB HL) in the poorer ear at baseline (T0) and follow-up (T1 aided), across all participants. Each line represents one subject, illustrating the change in hearing threshold following cochlear or bone conduction implantation. (**b**) Individual PTA4 thresholds (dB HL) in the better ear of AHL patients at baseline (T0) and follow-up (T1, best aided condition).

Figure 1a illustrates the individual evolution of PTA4 thresholds (poorer ear) from baseline (T0) to follow-up (T1 aided) after auditory implantation. The figure separates children and adults to provide a clearer view of hearing outcomes by age group. Each line represents one subject’s change in PTA4 thresholds, reflecting the degree of functional gain achieved. All patients showed substantial improvements in the aided condition (T1), with consistently lower PTA4 values compared to the unaided baseline. Figure 1b shows the evolution of PTA4 thresholds in the better ear for AHL subjects, measured at baseline (T0, unaided) and at follow-up (T1, in the best aided condition). At T1, most individuals were assessed while using a hearing aid in the better ear, except for one who was evaluated while unaided. These results underline the importance of optimal hearing aid fitting and regular use in patients with AHL undergoing auditory rehabilitation.

**Figure 2:**
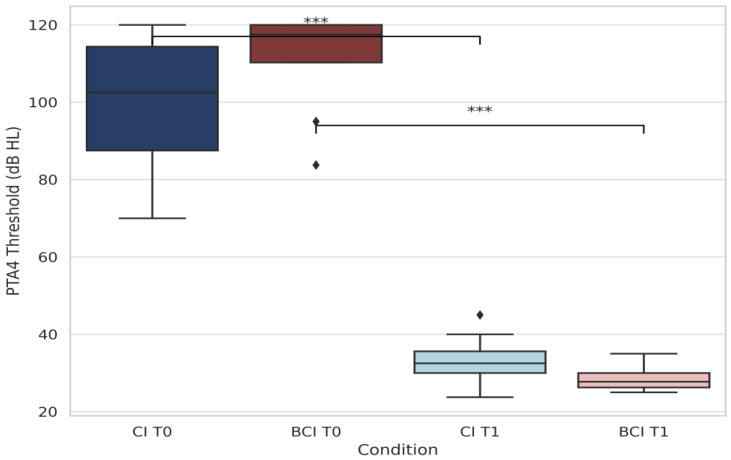
Comparative analysis of PTA4 thresholds (CI vs. BCI users). Boxplot showing PTA4 thresholds in the poorer ear at T0 and T1 for the CI and BCI groups, separately. Both CI (navy/light blue) and BCI (dark red/light coral) users showed statistically significant improvement post-implantation. Asterisks show significance: *** *p* < 0.001. Diamonds represent outliers.

Figure 2 provides a comparative analysis of PTA4 thresholds between CI and BCI users and quantitatively compares these groups. Statistically significant improvements were seen for both CI (*p* < 0.001) and BCI (*p* = 0.0002) groups (Wilcoxon signed-rank test). Postoperative thresholds achieved approximately 30 dB HL, confirming substantial auditory rehabilitation success in both implant types, with notably stronger results in CI users. The variation in individual responses suggests differences in baseline hearing, auditory adaptation, and amplification effects.

#### 3.2.2. Localization Accuracy (RMS)

Localization accuracy was evaluated using the root mean square (RMS) error in degrees across three listening conditions: unaided baseline (T0), unaided follow-up (T1 unaided), and aided follow-up (T1 aided). The RMS value reflects the average angular deviation between the presented and perceived sound source locations, with lower values showing better spatial hearing.

To assess within-subject improvements, statistical comparisons were performed using paired *t*-tests or Wilcoxon signed-rank tests, depending on normality. Normality was assessed using the Shapiro–Wilk test. Cohen’s d was calculated to find effect sizes, and power analyses were conducted to evaluate the robustness of the findings.

Across the full cohort (excluding n = four cases with missing T0 data), localization improved significantly from T0 (mean = 77.9° ± 13.8°) to T1 aided (mean = 44.6° ± 18.1°), as confirmed with a paired t-test (*p* < 0.001, d = 1.176, power = 0.9998). These results show a large and reliable improvement in spatial hearing following auditory implantation.

CI users showed consistent and substantial improvements, while BCI users showed more variable or modest improvements. These differences were further supported by statistical tests conducted within each subgroup.

**Figure 3:**
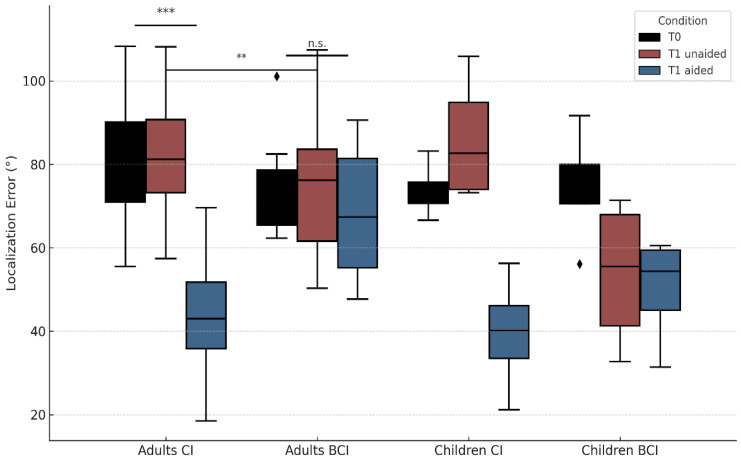
Localization performance across conditions. Boxplots showing RMS localization error in four subgroups (Adults CI, Adults BCI, Children CI, Children BCI) across three listening conditions: baseline (T0), T1 unaided, and T1 aided. Diamonds represent outliers. Asterisks indicate statistical significance within or between groups (** *p* < 0.01, *** *p* < 0.001). “n.s.” indicates non-significant differences.

Among adult CI users, RMS T0 was 81.9° ± 15.8° (55.5–108.3°), and RMS T1 aided was 43.7° ± 13.5° (18.5–69.6°), showing a significant improvement. Among adult BCI users, RMS T0 was 74.6° ± 12.1° (62.3–101.1°), while RMS T1 aided was 69.2° ± 16.3° (47.7–90.6°). Wilcoxon signed-rank tests revealed statistically significant improvements in CI users between T0 and T1 aided (z = 4.029, *p* < 0.01) and between T1 unaided and T1 aided (z = 4.180, *p* < 0.01). Among the pediatric recipients, significant improvements were seen both from T0 to T1 aided (median: 74.3° → 44.8°, *p* = 0.016) and from T1 unaided to T1 aided (*p* = 0.012), as confirmed by Wilcoxon tests. These findings were consistent across children with both CIs and BCIs, reinforcing the positive impact of early auditory implantation on spatial hearing development. No significant changes were seen in BCI users (T0 vs. T1 aided: *p* = 0.255; T1 unaided vs. T1 aided: *p* = 0.190). Localization performance across the four subgroups—adult CI users, adult BCI users, children with CIs, and children with BCIs—is shown in Figure 3. While statistically significant improvements were observed in adult CI users and in the pooled pediatric group, subgroup analyses in pediatric CI and BCI recipients did not reach significance individually, likely due to the limited sample size in each category (n < 5).

**Figure 4:**
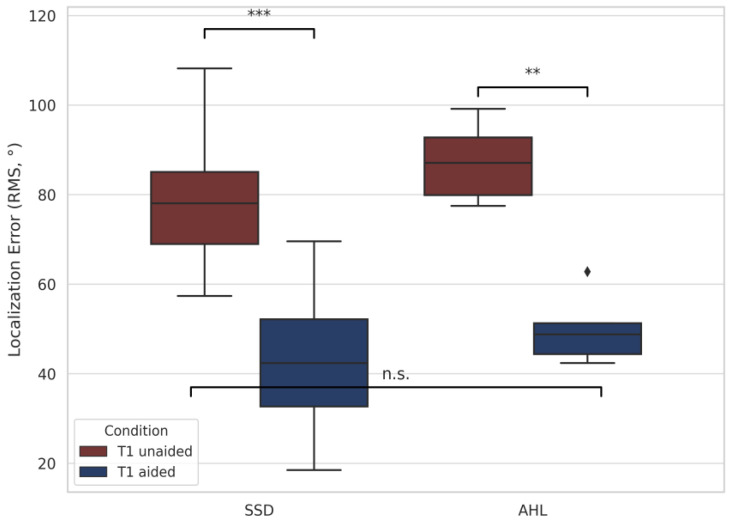
Boxplots of localization error (RMS, °) at follow-up unaided (T1), and with the device activated (T1 aided) in cochlear implant users with single-sided deafness (SSD) and asymmetric hearing loss (AHL). Outliers are represented by black diamonds (♦), defined as values outside 1.5 times the interquartile range Asterisks indicate statistical significance within or between groups (** *p* < 0.01, *** *p* < 0.001), “n.s.” indicates non-significant differences.

Figure 4 compares RMS performance in adult CI users with SSD versus CI users with AHL. SSD users showed significant improvements from T0 to T1 aided (*p* < 0.001) and from T1 unaided to T1 aided (*p* < 0.001). No statistically significant difference was found between SSD and AHL users at T1 aided (*p* = 0.197). This may reflect genuinely similar final performances or could be due to the limited sample size. Although AHL users did not show statistically significant changes across conditions, their mean RMS values followed a similar pattern of reduction.

The analysis of onset type among adult BCI users showed similar mean RMS values at T1 aided between perilingual and postlingual cases (t = 0.23, *p* = 0.823, d = –0.17; Mann–Whitney U = ns). However, due to the small sample size in this subgroup, the statistical power was low, and the absence of a significant difference should not be interpreted as evidence of no effect. These observations remain exploratory and do not allow firm conclusions regarding the influence of onset timing on localization outcomes in BCI users.

**Figure 5:**
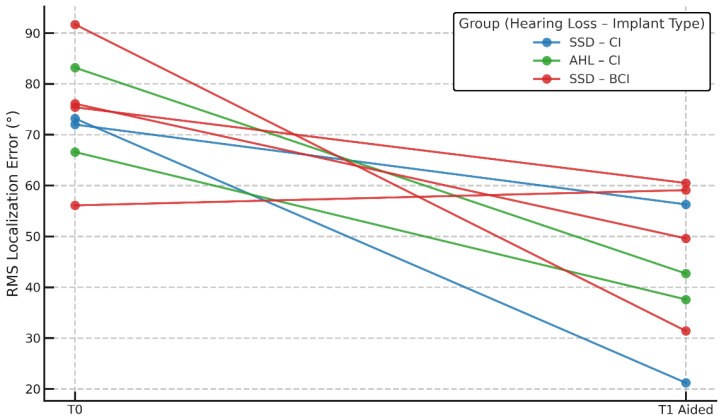
Individual localization outcome from baseline to follow-up in children. Line plot showing RMS localization error at baseline (T0) and follow-up (T1 aided) for each subject, separated by implant type. Most subjects demonstrated improved localization accuracy at follow-up. One BCI user exhibited a higher RMS error at T1, indicating a deterioration in spatial performance.

Figure 5 further illustrates individual trajectories in children by hearing loss type and device. Subjects showed improved RMS error post-implant, with some variability, supporting the notion that auditory implants help children regardless of etiology or device type.

Together, these results prove that cochlear implants offer large and statistically robust improvements in localization, particularly in adults, while BCI users showed less consistent gains.

#### 3.2.3. Speech Perception in Noise

Statistical comparisons were performed using Welch’s *t*-tests for independent group comparisons (e.g., SSD patients vs. AHL patients, CI users vs. BCI users), and one-sample *t*-tests were performed for within-group comparisons against zero improvement. Significance was set at *p* < 0.05. Post hoc effect sizes (Cohen’s d) and statistical power (1–β) were computed for each comparison to assess the robustness of observed effects. No corrections for multiple comparisons were applied, as the post hoc tests were hypothesis-driven.

Across the entire cohort, the mean binaural effects were as follows: head shadow effect = 4.7 ± 3.1 dB (range: −1.8 to 10.8), release from masking = 0.6 ± 3.2 dB (range: −6.0 to 6.6), binaural squelch = 1 ± 3.6 dB (range: −9.8 to 8.4), and binaural summation = 2.5 ± 3.7 dB (range: −2.4 to 12.6). One-sample *t*-tests confirmed significant improvements for head shadow (*p* < 0.001, d = −0.54, power = 0.99) and binaural summation (*p* < 0.001, d = 0.69, power = 0.99), while release from masking and binaural squelch did not reach significance.

When subgroup analysis was performed, CI SSD adults showed a head shadow effect of 4.9 ± 4 dB and binaural summation of 2.8 ± 3.6 dB, while adult BCI users showed scores of 3.8 ± 2.5 dB and 1.89 ± 2.92 dB, respectively. In children, all effects were positive, with notable improvements in head shadow (4.6 ± 3.1 dB), release from masking (3.3 ± 2.6 dB), and binaural squelch (2.7 ± 3 dB).

Although all binaural effects were numerically positive in each subgroup, none of the one-sample *t*-tests reached statistical significance (all *p* > 0.05). Calculated power values ranged from 0.05 to 0.33, underscoring the limited statistical strength of these subgroup analyses. Therefore, while the observed values are consistent with potential benefit, the current data do not permit reliable confirmation at the subgroup level.

**Figure 6:**
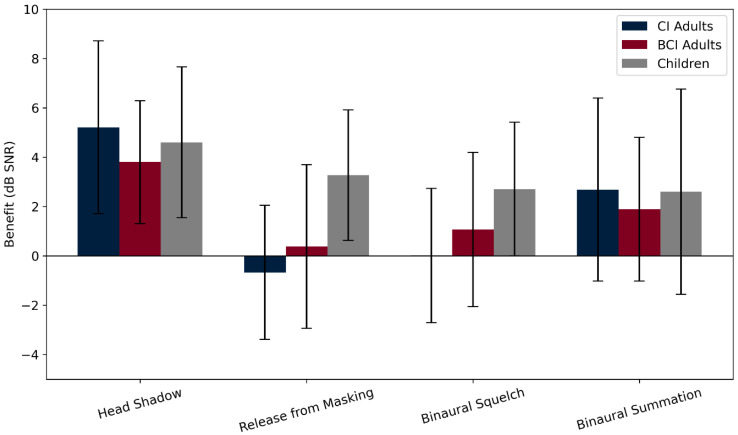
Binaural effects by group. The boxplots illustrate the distribution of four binaural effects—head shadow effect, release from masking, binaural squelch, and binaural summation—across three groups: CI SSD adults, BCI adults, and children. Each effect is expressed as the SRT improvement in the dB signal-to-noise ratio SNR (positive values indicate auditory benefit). The horizontal reference line at 0 dB denotes no change. Boxes represent interquartile ranges (IQRs), whiskers extend to 1.5 × IQR.

No significant differences were seen between adult CI and adult BCI users for any of the four effects (all *p* > 0.4). These findings are summarized in Figure 6, which displays the distribution of all binaural effects across groups. Notably, comparisons between binaural effects in the overall cohort revealed that the head shadow effect was significantly stronger than all other effects (all *p* < 0.01), having the largest mean improvement and highest statistical power (d = –0.54, power = 0.99). Head shadow consistently appeared as the most effective binaural mechanism in CI, BCI, and pediatric users alike. The binaural squelch effect was present in some individuals but was less consistent overall, with the group-level gains being smaller and more variable.

**Figure 7:**
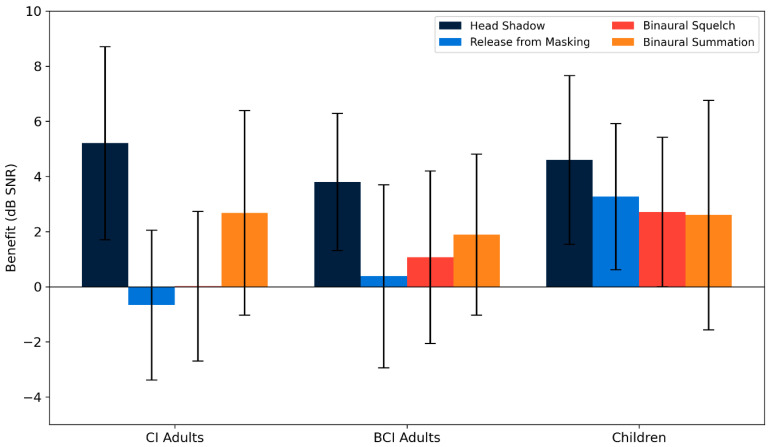
Binaural benefit patterns within groups. Bar plot showing the average improvement in speech reception threshold (SRT, in dB signal-to-noise ratio) for each of the four binaural effects—head shadow, release from masking, binaural squelch, and binaural summation—within each participant group: adults with CI, adults with BCI, and children. Each bar shows the mean benefit with standard deviation. The head shadow effect consistently appeared as the most prominent binaural advantage across all groups, as also confirmed statistically in the main cohort (*p* < 0.01).

The variability within each group was substantial for all effects, as reflected in the large standard deviations (Figure 7). These differences likely reflect both subject-specific characteristics and differences in device performance and auditory adaptation. On average, children showed the greatest overall binaural benefit, particularly in the squelch and summation conditions, while CI SSD adults showed a strong head shadow advantage, but minimal benefit in central binaural integration.

**Figure 8:**
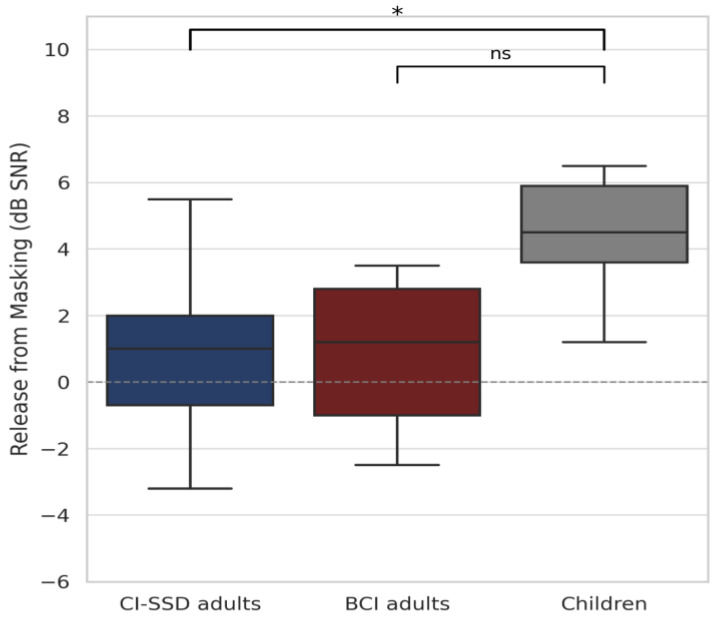
Release from masking by group. Children showed significantly greater release from masking compared to CI-SSD adults (t = 3.04, *p* = 0.007). The difference between children and adult BCI users did not reach statistical significance (t = 2.10, *p* = 0.059). Values are expressed in dB SNR, with higher values indicating better ability to extract speech from spatially separated noise. Asterisks indicate statistical significance within or between groups (* *p* < 0.05), “n.s.” indicates non-significant differences.

Statistical comparisons between groups (Figure 8) revealed that children performed significantly better than CI SSD adults for release from masking (t = 3.04, *p* = 0.007, d = 1.54). The difference with adult BCI users did not reach statistical significance (t = 2.10, *p* = 0.059, d = 1.14), although mean values remained higher in the pediatric cohort. While this finding may reflect an enhanced capacity to extract speech from spatially separated noise, it should be interpreted with consideration of the key clinical differences between groups. The pediatric group included both CI and BCI users, with either congenital/perilingual or acquired hearing loss. Nonetheless, due to earlier implantation, even children with congenital or early-onset deafness experienced significantly shorter durations of auditory deprivation compared to adults. In contrast, several adult BCI users had long-standing perilingual or postlingual deafness, potentially limiting the plasticity of their auditory pathways. These differences likely contributed to the observed advantage in pediatric performance.

A comparative analysis of adult CI users with SSD versus AHL was also conducted using Welch’s *t*-tests. No statistically significant differences were found (head shadow: t = −0.752, *p* = 0.463, d = −0.27; release from masking: t = 0.887, *p* = 0.394, d = 0.39; binaural squelch: t = 0.440, *p* = 0.673, d = 0.23; binaural summation: t = 0.177, *p* = 0.865, d = 0.10). Power analyses for these comparisons were low (range: 0.05–0.11), suggesting limited ability to detect differences. Despite this, the results suggest that AHL patients may experience comparable binaural benefits following cochlear implantation, reinforcing the relevance of CI for both SSD and AHL profiles.

#### 3.2.4. Tinnitus Severity

Tinnitus severity was evaluated in adult patients (≥18 years) using a visual analog scale (VAS, 0–10). To reflect the overall impact of auditory implants on the tinnitus burden in the population, all adults were included in the analysis, regardless of baseline tinnitus presence. Only T0 and T1 aided values were considered, excluding T1 unaided due to lack of control over implant-off duration.

Among cochlear implant (CI) users (n = 18), tinnitus severity decreased from a mean of 5.67 at T0 to 1.78 at T1 aided. A Wilcoxon signed-rank test confirmed a significant reduction (*p* = 0.0001, r = 0.71), with a high effect size and statistical power (1–β = 0.96). In the BCI group (n = 9), tinnitus scores decreased from 1.89 to 0.56 (mean reduction 1.6 ± 0.9), but this change was not statistically significant (*p* = 0.25, r = 0.50, power ≈ 0.30).

**Figure 9:**
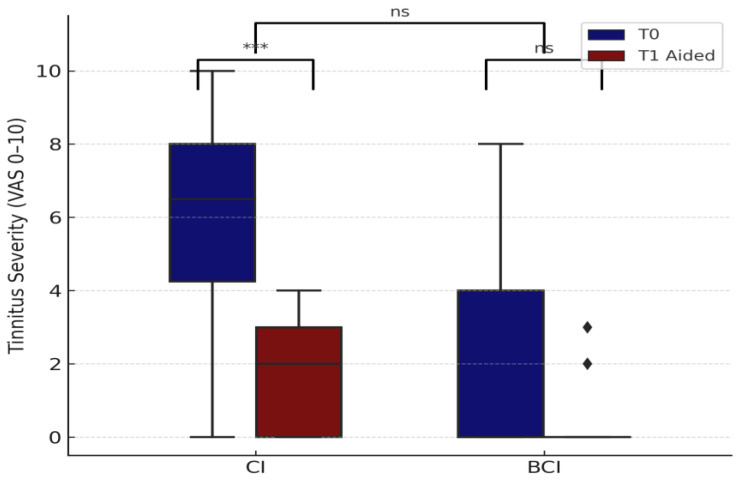
Tinnitus severity before and after implantation in adult users of CIs and BCIs. The boxplots show tinnitus severity at baseline (T0) and at follow-up with the device activated (T1 aided). Diamonds represent individual values. Asterisks indicate statistically significant differences (*** *p* < 0.001); ns = not significant.

Although CI users showed a greater mean reduction (2.8 ± 2.0), a Mann–Whitney test comparing tinnitus improvement between CI and BCI users did not reach significance (*p* = 0.371) (Figure 9). Importantly, no new cases of tinnitus were seen after implantation, and all individuals with perilingual onset (n = 4) consistently reported VAS = 0 at both time points. This suggests that the tinnitus burden in this population was restricted to postlingual onset.

A subgroup comparison between CI users showed no significant difference in tinnitus improvement between AHL and SSD cases (*p* = 0.7872), indicating that tinnitus relief is similarly achievable in both types of asymmetric hearing loss.

These findings highlight the role of cochlear implants in alleviating tinnitus burden in adults, particularly among those with pre-existing symptoms. While BCI users showed a favorable trend, the effect was not statistically robust, and interpretation is limited by sample size. Clinically, tinnitus was typically reported in the poorer-hearing or deaf ear, especially in SSD patients, which is consistent with the focus of our analysis on the implant-aided side. In AHL cases, bilateral or diffuse tinnitus was described more often. However, tinnitus laterality was not coded systematically and was therefore not included as a variable in the analysis.

#### 3.2.5. Self-Reported Hearing Outcomes—SSQ12

Self-reported hearing outcomes were assessed using the SSQ12A at baseline and SSQ12B/C to evaluate perceived benefit after implantation. Among adults, baseline SSQ12A scores were slightly higher in BCI users (5.2 ± 2.1) compared to CI users (4.5 ± 2.1), though the difference was not statistically significant (*p* = 0.37). Within the adult CI group, SSD patients reported higher initial scores than AHL patients (4.8 ± 1.9 vs. 3.5 ± 2.5), but this difference also did not reach statistical significance (*p* = 0.32). In children, baseline SSQ12A scores were comparable between CI and BCI users, with no significant differences.

Following auditory rehabilitation, all participants reported significant perceived improvements across the SSQ12B/C scale. Mean total improvement scores were 1.7 ± 0.9 in adult CI users, 1.4 ± 0.5 in adult BCI users, and 1.9 ± 0.5 in children, with pediatric participants reporting the highest overall benefit. The difference between children and adult BCI users was statistically significant (*p* = 0.0035, Bonferroni-corrected *p* = 0.0105), while the comparisons between children and adult CI users, and between adult CI users and adult BCI users, were not significant.

**Figure 10:**
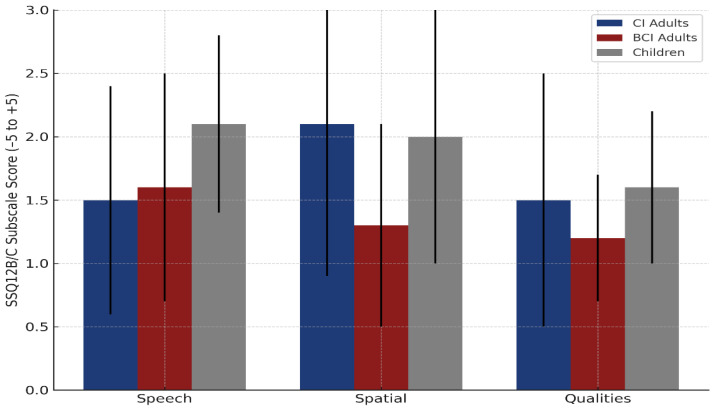
SSQ12B/C subscale improvements following auditory rehabilitation. Bar chart showing mean perceived improvement (± SD) on the SSQ12B/C subscales (speech, spatial, qualities) across three groups: adults with a cochlear implant (CI), adults with a bone conduction implant (BCI), and pediatric users. All groups reported subjective benefits from implantation.

To further explore the perceived benefit of implantation, SSQ12B/C subscale scores were analyzed separately for the three domains: speech, spatial, and qualities. Figure 10 illustrates these improvements across adult CI users, adult BCI users, and pediatric users. In the speech subscale, children reported the greatest gains (2.1 ± 0.7), followed by adult BCI users (1.6 ± 0.9) and adult CI users (1.5 ± 0.9). No significant difference was observed between adult CI and BCI users (*p* = 0.8808, Bonferroni *p* = 1.000). In the spatial subscale, adult CI users showed the largest benefit (2.1 ± 1.2), closely followed by children (2.0 ± 1.0), while adult BCI users reported lower gains (1.3 ± 0.8). The difference between adult CI and BCI users approached statistical significance (*p* = 0.0547, Bonferroni *p* = 0.1641). For sound qualities, children again reported the highest benefit (1.6 ± 0.6), followed by adult CI users (1.5 ± 1.0) and adult BCI users (1.2 ± 0.5), with no significant difference between adult groups (*p* = 0.2439, Bonferroni *p* = 0.7318).

#### 3.2.6. Correlations and Device Use

Although not the focus of this study, we explored correlations between key clinical outcomes and individual characteristics such as daily device use and the duration of deafness. These analyses were conducted to gain a better understanding of the variability in patient response within our initial cohort of individuals with asymmetric or unilateral hearing loss.

Daily device use varied by age and implant type. These values represent the total daily time the implant was worn and powered on, as reported by the patients, regardless of environmental sound levels. Therefore, “device use” in this context should be interpreted as a general indicator of use rather than a measure of auditory input or sound exposure. CI users generally reported longer daily use compared to BCI users. Among adults with a CI, the mean daily use was 7.7 h (range: 1.5–12.0), while children with a CI showed the highest usage (mean: 8.0 h/day; range: 2.0–12.0). BCI users reported lower use, with adult BCI users averaging 5.3 h/day (range: 1.0–10.0), and children with BCI averaging 7.5 h/day (range: 2.0–12.0). These patterns suggest that younger users, especially those with a CI, tend to maintain more consistent auditory stimulation, which is likely supported by structured rehabilitation programs and caregiver involvement.

A significant negative correlation was found between RMS localization error in the aided condition and SSQ12B/C spatial scores (r = –0.35, *p* = 0.037), indicating that better localization accuracy is associated with greater perceived spatial benefit. Additionally, improvement in RMS performance (T1 unaided–T1 aided) positively correlated with the full SSQ spatial score (r = 0.432, *p* = 0.009), reinforcing the relationship between objective gains and subjective experience of spatial hearing.

Daily use of the auditory device was positively correlated with overall SSQ12B/C improvement (r = 0.569, *p* < 0.001), as well as with each subscale: speech (r = 0.45, *p* = 0.006), spatial (r = 0.41, *p* = 0.013), and qualities (r = 0.50, *p* = 0.002). These associations highlight the role of consistent auditory stimulation in enhancing self-reported hearing outcomes. A significant correlation was observed between longer durations of deafness and higher RMS error in the aided condition (r = 0.44, *p* = 0.007), suggesting that prolonged auditory deprivation may negatively impact localization ability. However, no significant correlation was found between daily use and RMS T1 aided performance (r = –0.23, *p* = 0.17), indicating that while consistent use is beneficial, it may not fully compensate for deficits related to auditory deprivation.

Daily device use also correlated moderately with tinnitus reduction (r = 0.42, *p* = 0.011), while it was inversely correlated with the duration of deafness (r = –0.476, *p* = 0.003). These findings support the hypothesis that earlier and consistent use may facilitate tinnitus relief, possibly through auditory masking or neuroplastic mechanisms.

Binaural squelch was the only speech-in-noise parameter that significantly correlated with the SSQ12 scores, showing strong associations with both the SSQ12B/C total score (r = 0.585, *p* = 0.0002) and the speech subscale (r = 0.505, *p* = 0.0017). This suggests that patients who derive greater benefit from binaural squelch also report enhanced speech understanding and overall hearing improvement. In contrast, other binaural measures, such as head shadow, release from masking, and binaural summation, did not show statistically significant correlations with SSQ12 scores.

ANCOVA analyses showed no significant influence of age or device use on head shadow effect, release from masking, or binaural summation. However, daily device use significantly predicted binaural squelch performance (*p* = 0.048), suggesting that auditory experience may enhance spatial filtering capabilities. A regression model for adult CI users indicated a trend-level effect of daily use on RMS T1 aided scores (β = –2.05, *p* = 0.07), explaining 33.1% of the variance (R² = 0.331, *p* = 0.0218). The duration of deafness was not a significant predictor in this model (β = 0.48, *p* = 0.098). Bootstrap analysis for subjects using their device ≥8 h/day yielded an estimated mean RMS T1 aided of 50.2° (95% CI: 41.2–59.6°), indicating moderate spatial accuracy with high usage. To better contextualize this result, we examined the baseline and unaided scores in the same subgroup (n = 15). The mean RMS error was 82.8° ± 14.1° at baseline (T0), 82.7° ± 13.9° at T1 unaided, and 50.3° ± 19.7° at T1 aided, with an average unaided-to-aided gain of 32.5° ± 21.6°. When stratified by device, CI users (n = 11) showed substantial benefit (mean improvement = 41.4° ± 17.1°), while BCI users (n = 4) showed minimal change (7.9° ± 10.3°), suggesting that the group-level accuracy was primarily driven by CI users.

**Figure 11:**
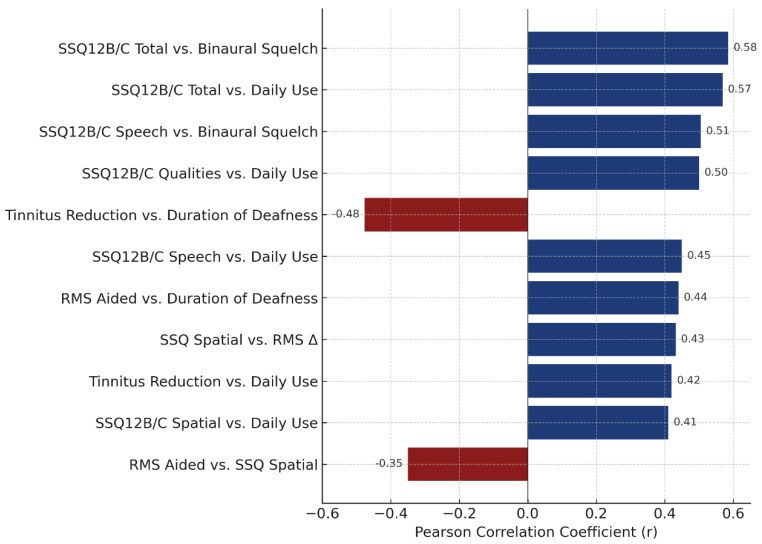
Correlations between auditory outcomes and patient characteristics.

These findings collectively emphasize the complex interplay between auditory experience, perceptual outcomes, and residual neural plasticity (Figure 11). Among the binaural effects, binaural squelch demonstrated the strongest link between objective performance and subjective benefit, suggesting that it may represent the most functionally relevant marker of binaural integration in this population.

Horizontal bar plot displaying Pearson correlation coefficients (r) for statistically significant relationships (*p* < 0.05) between clinical and auditory outcomes. Positive correlations are shown in dark blue (e.g., consistent device use and SSQ12 subscales), while negative correlations are shown in burgundy (e.g., the duration of deafness and localization error). The strongest associations were observed between binaural squelch and both the SSQ12 total and speech subscale scores, highlighting its functional relevance in patient-reported benefit.

## 4. Discussion

This study aimed to evaluate the outcomes of auditory rehabilitation in individuals with SSD and AHL following cochlear implantation or bone conduction implantation, based on standard clinical indications. We focused on improvements in hearing thresholds, localization, speech-in-noise performance, tinnitus relief, and subjective hearing experience, while examining the influence of patient-specific variables and binaural processing effects.

Improvements in PTA4 thresholds in the implanted ear confirmed restored access to sound for both CI and BCI users. AHL patients also showed minor improvements in their better ear, likely due to the adjusted contralateral hearing aid settings, highlighting the importance of bilateral management. These findings are consistent with prior reports on auditory restoration in asymmetrical hearing loss.

Localization improved significantly post-implantation, especially in CI users. The RMS localization error dropped across the CI users. This aligns with previous studies showing RMS gains of 25–35° following the use of CIs by SSD patients [15,21], although outcomes rarely reach normal-hearing accuracy [20] (Oh et al. Randomized controlled trials have similarly confirmed CIs’ superiority over BCD and CROS solutions for spatial hearing restoration [24,40]).

BCI users, particularly adults, demonstrated smaller and more variable spatial gains. Among adult CI users, the SSD and AHL subgroups achieved comparable aided RMS values (~44°), though only SSD users improved significantly across all listening conditions, supporting previous findings [45]. Children, regardless of implant type, showed consistent improvement in localization, with average RMS reductions of ~30°. This may reflect neuroplastic compensation in the developing auditory system, even in the absence of formal training [32,34].

The duration of deafness correlated with poorer localization outcomes, reinforcing the value of early intervention [37]. A subgroup of high-use CI recipients (≥8 h/day) showed better aided localization (mean RMS = 50.2°), suggesting a possible dose–response relationship between daily stimulation and spatial accuracy. However, this pattern should be interpreted with caution given the small sample size and lack of stratification shown in Figure 4, which reflects group-level averages rather than usage-based subgroups. Importantly, daily use alone did not significantly correlate with aided RMS scores, indicating that consistent exposure may support—but not guarantee—spatial adaptation. These observations align with evidence emphasizing the role of early and sustained auditory input in optimizing spatial hearing outcomes (Santopietro et al., 2024) [33].

From a clinical perspective, localization is one of the most functionally relevant auditory abilities, contributing to sound awareness, safety, and communication in complex environments. The restoration of this ability through cochlear implantation confirms that spatial hearing is not only achievable, but also generalizable across different user groups, especially when intervention is timely. The discrepancy between CI and BCI outcomes, particularly in adults, underlines the limitations of passive acoustic transmission in supporting the encoding of spatial cues and stresses the importance of tailoring device selection to auditory goals. In children, the results suggest that developmental neuroplasticity may support more flexible integration of auditory input, potentially bypassing some of the mechanical limitations associated with BCIs.

Binaural effect analysis revealed that head shadow was the most consistent benefit across groups. This effect is driven by simple acoustic separation, based on the attenuation of sound intensity as it travels around the head from one ear to the other, rather than by true binaural integration. As such, it can be accessed regardless of age or device type. These results are in line with previous reports emphasizing that head shadow is an acoustic phenomenon largely independent of neural synchrony [27].

Binaural summation showed modest but consistent improvements, suggesting that access to bilateral stimulation provides perceptual advantages even in asymmetric listeners. Binaural squelch, which depends more on central auditory integration, was present only in select individuals and reached notable levels, primarily in children. A moderate-to-strong correlation between binaural squelch and SSQ12B/C scores (r > 0.5) further supports its relevance in subjective spatial benefit, in line with prior observations [21]. These results are consistent with multicenter studies reporting large interindividual variability in binaural integration following CI use (Marx et al., 2021; Wesarg et al., 2024) [27,28].

Release from masking demonstrated the most group-dependent variability. While children and BCI users showed positive effects, CI-SSD users exhibited no measurable benefit. This likely reflects reduced access to spatial unmasking cues in postlingually implanted CI users, possibly due to mismatched input timing, spectral differences, or insufficient binaural experience. The significant difference observed between children and CI SSD users (*p* = 0.007) underscores the importance of early auditory rehabilitation and greater plasticity during development.

Quantitative comparisons showed that CI SSD adults had the strongest binaural effects. Adult BCI users showed lower mean values for head shadow and summation, and limited benefit in summation and squelch—likely due to the lack of synchronous neural input between bone and air conduction pathways. Children showed positive values across all four effects, with notable improvements in head shadow, release from masking, and binaural squelch, reflecting meaningful perceptual gains during development.

Despite these findings, one-sample *t*-tests did not reach statistical significance within any subgroup (all *p* > 0.05), and power values ranged from 0.05 to 0.33, indicating limited ability to confirm the observed effects statistically. These findings align with previous studies reporting high interindividual variability in binaural outcomes [27,28]. No significant differences were observed between SSD and AHL adults with CIs for any of the four binaural effects (head shadow: *p* = 0.463; summation: *p* = 0.865; squelch: *p* = 0.673; release from masking: *p* = 0.394). While statistical power was low, the similarity in average values supports the hypothesis that cochlear implantation offers comparable binaural benefits for both asymmetry profiles.

ANCOVA revealed that neither age nor daily device use significantly influenced head shadow, summation, or release from masking. However, daily use was associated with greater binaural squelch (*p* = 0.048), suggesting that auditory training and consistent device exposure may enhance central binaural integration.

While no significant correlation was found between daily device use and aided RMS performance (r = –0.23, *p* = 0.17), clinical guidelines, such as those proposed by Dillon et al., suggest that consistent use of 8 h or more per day is associated with more effective auditory rehabilitation. This threshold was used in our bootstrap analysis. In comparison, Roger et al. [49] proposed a slightly higher threshold of 9 h/day when examining differences in speech-in-noise outcomes, which may indicate a gradual continuum rather than a fixed boundary. Although the current study did not stratify patients based on that specific cut-off due to sample size limitations, our results support the idea that more consistent use may enhance certain auditory outcomes, particularly binaural integration, as reflected in the significant association with binaural squelch. Future studies with larger samples should further examine the relationship between usage intensity and spatial or speech-in-noise performance.

The tinnitus analysis, which was restricted to adults, demonstrated significant suppression with device activation. CI users showed greater average reduction than BCI users, particularly in SSD with postlingual onset. This improvement was likely due to the cochlear input reversing central hyperactivity [20,50]. A moderate correlation between daily use and tinnitus relief further supports the value of consistent stimulation. These results align with systematic reviews reporting meaningful and sustained tinnitus suppression following cochlear implantation [18,21]. While speculative, these findings may suggest a neuromodulatory effect dependent on afferent stimulation and device type. Further research is needed to clarify the underlying mechanisms and to identify predictors of tinnitus relief across different clinical profiles.

Subjective hearing improvements, assessed via SSQ12, were substantial. This correlation should be interpreted in light of the prior literature. Blamey et al. [37] proposed a staged model of auditory performance decline, emphasizing that outcomes are shaped by both the duration and severity of hearing loss [37]. In addition, studies such as that by An et al. [51] indicate that prior auditory stimulation—even when inconsistent—may help preserve auditory processing and improve post-implantation outcomes. However, given that most patients in our cohort had little or no consistent history of hearing aid or CROS use, it is likely that they did not benefit from such protective effects, which may partly explain the observed relationship between deafness duration and spatial accuracy. The SSQ12B/C total improved, with the greatest gains observed in speech understanding, followed by sound quality and spatial perception. CI users showed greater gains in spatial subscales than BCI users, consistent with superior spatial resolution via electrical stimulation [39,40]. Children reported the largest gains in speech understanding, reflecting age-related neural plasticity (Santopietro et al., 2024; Benchetrit et al., 2021) [33,34].

Importantly, subjective outcomes correlated with objective measures, including binaural squelch and localization, affirming the real-life relevance of these effects. Daily device use was positively associated with SSQ12 improvements, supporting the need for active engagement. These findings emphasize the psychosocial and functional value of auditory rehabilitation, particularly when personalized and initiated early. The alignment between patient-reported outcomes and behavioral improvements has been confirmed in multiple trials investigating CI effectiveness in SSD (Sydlowski et al., 2022; Oh et al., 2023) [20,25].

Correlation and regression analyses provided additional insights. SSQ12B/C spatial scores negatively correlated with RMS error in the aided condition (r = −0.35) and positively with RMS improvement (r = 0.43), confirming the link between spatial hearing accuracy and subjective benefit. Daily device use showed significant positive correlations with overall SSQ12B/C improvement (r = 0.57) and with each subscale (speech: r = 0.45; spatial: r = 0.41; qualities: r = 0.50). Tinnitus relief also correlated with daily use (r = 0.42), while the duration of deafness was negatively associated with it (r = −0.48), reinforcing the importance of early and consistent auditory input.

Binaural squelch demonstrated the strongest relationship with SSQ12 spatial perception (r > 0.5), underscoring its clinical relevance. Regression analysis in adult CI users showed that daily use explained 33.1% of variance in the aided RMS scores, though the effect was marginal (*p* = 0.07). The duration of deafness did not significantly predict outcomes. These observations, though exploratory, suggest that while certain auditory outcomes are influenced by individual use patterns, others depend more on timing and the duration of sensory deprivation.

These correlations were not the focus of this study and should be interpreted cautiously, particularly given the cohort size and observational design. Nonetheless, they support emerging evidence on the role of patient-specific variables in shaping auditory rehabilitation outcomes and offer valuable directions for further investigation.

### 4.1. Study Strengths

This study provides clinical evidence about auditory rehabilitation with cochlear implants and bone conduction devices. The longitudinal design and inclusion of unselected patients strengthen its clinical relevance, ensuring that results reflect real-world auditory outcomes.

### 4.2. Limitations and Potential Biases

Several limitations and potential sources of bias should be considered when interpreting these findings. This study was conducted in a single tertiary referral center, which may limit the generalizability of results to broader clinical settings. Small sample sizes, particularly in the pediatric and BCI subgroups, reduce statistical power and increase the likelihood of type II error. Clinical heterogeneity in etiology, onset age, and device type (CIs vs. BCIs) may have influenced outcomes. Although subjective outcome measures such as SSQ12 and tinnitus ratings were collected using validated tools, their reliance on self-reporting introduces potential response bias. The SSQ12 questionnaire was formally validated in Romanian, while tinnitus severity was assessed via a VAS scale, which—though conceptually validated—is not based on a standardized tinnitus-specific inventory. Furthermore, unaided assessments at T1 may have been affected by uncontrolled variation in implant-off durations, introducing potential measurement bias. While clinical history of prior hearing aid user or CROS use was collected, it was not used as a grouping variable in the analysis. Observer bias was minimized through the use of standardized test protocols, but cannot be entirely excluded. These limitations underscore the need for cautious interpretation, especially when comparing subgroups with differing clinical profiles. Nonetheless, statistically significant improvements were demonstrated in core auditory domains, supporting the clinical relevance of the findings in real-world rehabilitation scenarios [37,49,51].

Finally, the observational design of this study inherently limits causal inferences. All implant decisions were made by the clinical team based on national program criteria and patient-specific factors, without randomization or blinding. Despite these limitations, statistically robust effects were observed in several key domains (e.g., localization, binaural squelch, SSQ12 improvement), supporting the overall consistency and relevance of the findings.

While many aspects of auditory rehabilitation—particularly regarding the efficacy of cochlear implantation in SSD and AHL—have been clarified through large-scale, multicenter studies, key questions remain. Future research should go beyond outcome validation and focus on establishing a deeper understanding of individual variability in spatial and binaural performance. This includes exploring how neural plasticity, age at onset, and the duration and type of auditory deprivation influence the extent of functional restoration.

Special attention should be given to the differential effects of CI and BCI systems on auditory integration, especially in pediatric and perilingual-onset populations, where neurodevelopmental dynamics may modulate outcomes in ways that are not yet fully understood. The interaction between residual hearing and device input and the role of structured auditory training are also promising avenues for optimization.

Finally, the growing interest in the neuromodulatory impact of implants—such as tinnitus suppression and perceptual recalibration—highlights the need for integrative research bridging clinical audiology, cognitive neuroscience, and patient-reported outcomes. Addressing these challenges will not only improve patient selection and rehabilitation protocols, but may also open new frontiers in auditory neuroscience.

## 5. Conclusions

This study demonstrates that both cochlear implants (CIs) and bone conduction implants (BCIs), when selected according to clinical criteria, offer measurable auditory benefits in cases of single-sided deafness and asymmetric hearing loss. CI users, especially adults, achieved superior improvements in spatial hearing and tinnitus suppression, while BCI users showed more variable gains with favorable outcomes, particularly in pediatric cases.

By analyzing real-world outcomes across distinct indication profiles, this study emphasizes the importance of individualized implant selection, accounting for anatomical constraints, onset timing, and the duration of deprivation. These findings support current clinical guidelines and underline the value of early and tailored intervention strategies.

In clinical practice, careful patient profiling remains key to optimizing the benefits of auditory implants, particularly in complex asymmetric hearing scenarios.

## Figures and Tables

**Table 1:** (**a**) Demographic characteristics of adult participants. (**b**) Demographic characteristics of pediatric participants.

(**a**)							
**Subject ID**	**Gender**	**Age at Implant (Years)**	**Etiology**	**Onset**	**Duration of Deafness (Years)**	**Electrode CI Type/BCI System**	**Follow-Up Period (Months)**
S1	F	52.0	trauma	postlingual	1.0	Cochlear Slim Straight	16.0
S2	M	18.0	viral infection	postlingual	8.0	MED-EL FLEX 28	16.0
S3	F	49.0	Scohl-vest	postlingual	2.0	MED-EL FLEX 28	12.0
S4	F	30.0	Scohl-vest	postlingual	4.0	MED-EL FLEX 28	18.0
S5	M	49.0	trauma	postlingual	15.0	Cochlear Slim Straight	16.0
S6	F	52.0	trauma	postlingual	2.0	Cochlear Slim Straight	14.0
S7	F	56.0	Scohl-vest	postlingual	8.0	Cochlear Slim Straight	14.0
S8	M	50.0	Scohl-vest	postlingual	5.0	Cochlear Slim Straight	16.0
S9	M	42.0	trauma	postlingual	2.0	MED-EL FLEX 28	14.0
S10	F	35.0	Scohl-vest	postlingual	4.0	MED-EL FLEX 28	12.0
S11	M	47.0	viral infection	postlingual	1.0	MED-EL FLEX 28	12.0
S12	F	33.0	Scohl-vest	postlingual	9.0	Cochlear Slim Straight	18.0
S13	M	33.0	schwannoma	postlingual	2.0	MED-EL FLEX 28	12.0
S14	F	47.0	Scohl-vest	postlingual	9.0	MED-EL FLEX 28	14.0
S15	F	54.0	otospongiozsis	postlingual	7.0	Cochlear Slim Straight	16.0
S16	F	45.0	otospongiozsis	postlingual	5.0	MED-EL FLEX 28	16.0
S17	F	52.0	Scohl-vest	postlingual	1.0	Cochlear Slim Straight	14.0
S18	F	23.0	ototoxicity	postlingual	13.0	Digisonic SP EVO	24.0
S19	M	63.0	viral infection	postlingual	25.0	Cochlear Baha 5	14.0
S20	F	37.0	labyrinthitis	postlingual	10.0	Cochlear Baha 5	16.0
S21	M	52.0	labyrinthitis	postlingual	3.0	Cochlear Baha 5	14.0
S22	M	51.0	trauma	postlingual	0.5	MED-EL Bonebridge	12.0
S23	M	35.0	unknown	perlingual	35.0	MED-EL Bonebridge	12.0
S24	M	54.0	unknown	perlingual	54.0	MED-EL Bonebridge	14.0
S25	F	25.0	unknown	perlingual	25.0	MED-EL Bonebridge	12.0
S26	M	30.0	unknown	perilingual	30.0	MED-EL Bonebridge	12.0
S27	F	20.0	unknown	perilingual	20.0	MED-EL Bonebridge	12.0
(**b**)							
**Subject ID**	**Gender**	**Age at Implant (Years)**	**Etiology**	**Onset**	**Duration of Deafness (Years)**	**Electrode Type CI/BCI System**	**Follow-Up Period (Months)**
S1	M	16.0	viral infection	postlingual	8.0	Cochlear Slim Straight	16.0
S2	M	13.0	unknown	postlingual	6.0	MED-EL FLEX 28	12.0
S3	F	12.0	unknown	postlingual	5.0	Digisonic SP EVO	24.0
S4	M	9.0	unknown	postlingual	6.0	MED-EL FLEX 28	12.0
S5	F	13.0	labyrinthitis	postlingual	2.0	MED-EL FLEX 28	16.0
S6	M	10.0	unknown	perilingual	10.0	MED-EL Bonebridge	12.0
S7	F	15.0	unknown	perilingual	15.0	MED-EL Bonebridge	12.0
S8	F	15.0	unknown	perilingual	15.0	MED-EL Bonebridge	14.0
S9	M	11.0	unknown	perilingual	11.0	MED-EL Bonebridge	14.0

Note: CI: cochlear implant; BCI: bone conduction implant.

## Data Availability

The data are not publicly available due to institutional restrictions but may be provided by the corresponding author upon reasonable request and with permission from the hosting institution.

## Abbreviations

AHL	Asymmetric hearing loss
BCI	Bone conduction implant
CI	Cochlear implant
CROS	Contralateral routing of signals
dB HL	Decibel hearing level
dB SNR	Decibel signal-to-noise ratio
eABR	Electrically evoked auditory brainstem response
eALR	Electrically evoked auditory late response
HL	Hearing loss
MRI	Magnetic resonance imaging
PTA	Pure-tone average
RMS	Root mean square (localization error)
SPN	Speech-in-noise
SRT	Speech reception threshold
SSD	Single-sided deafness
SSQ12	Speech, Spatial, and Qualities of Hearing Scale–12-item version
TIN	Tinnitus score
VAS	Visual analog scale
S_0_N_0_	Speech and noise presented frontally (0° azimuth)
S_0_N_AHL/SSD	Speech frontally, noise at impaired side (AHL/SSD)
S_AHL/SSD N_0_	Speech at impaired side, noise frontally
S_AHL/SSD N_NH	Speech at impaired side, noise at normal-hearing ear
NAH	Normal-acoustic hearing ear (used in spatial test notation)
